# Somatosensory dysfunction is masked by variable cognitive deficits across patients on the Alzheimer's disease spectrum

**DOI:** 10.1016/j.ebiom.2021.103638

**Published:** 2021-10-21

**Authors:** Alex I. Wiesman, Victoria M. Mundorf, Chloe C. Casagrande, Sara L. Wolfson, Craig M. Johnson, Pamela E. May, Daniel L. Murman, Tony W. Wilson

**Affiliations:** a McConnell Brain Imaging Centre, Montreal Neurological Institute, McGill University, 845 Sherbrooke St W, Montreal, QC H3A 0G4, Canada; b Department of Neurological Sciences, University of Nebraska Medical Center (UNMC), Omaha, NE, USA; c Center for Brain, Biology, and Behavior, University of Nebraska – Lincoln, Lincoln, NE, USA; d Institute for Human Neuroscience, Boys Town National Research Hospital, Boys Town, NE, USA; e Geriatrics Medicine Clinic, UNMC, Omaha, NE, USA; f Department of Radiology, UNMC, Omaha, NE, USA; g Memory Disorders and Behavioral Neurology Program, UNMC, Omaha, NE, USA

**Keywords:** Sensory gating, Amyloid-β, Magnetoencephalography, Gamma oscillations, Neuropsychology

## Abstract

**Background:**

Alzheimer's disease (AD) is generally thought to spare primary sensory function; however, such interpretations have drawn from a literature that has rarely taken into account the variable cognitive declines seen in patients with AD. As these cognitive domains are now known to modulate cortical somatosensory processing, it remains possible that abnormalities in somatosensory function in patients with AD have been suppressed by neuropsychological variability in previous research.

**Methods:**

In this study, we combine magnetoencephalographic (MEG) brain imaging during a paired-pulse somatosensory gating task with an extensive battery of neuropsychological tests to investigate the influence of cognitive variability on estimated differences in somatosensory function between biomarker-confirmed patients on the AD spectrum and cognitively-normal older adults.

**Findings:**

We show that patients on the AD spectrum exhibit largely non-significant differences in somatosensory function when cognitive variability is not considered (*p*-value range: .020–.842). However, once attention and processing speed abilities are considered, robust differences in gamma-frequency somatosensory response amplitude (*p* < .001) and gating (*p* = .004) emerge, accompanied by significant statistical suppression effects.

**Interpretation:**

These findings suggest that patients with AD exhibit insults to functional somatosensory processing in primary sensory cortices, but these effects are masked by variability in cognitive decline across individuals.

**Funding:**

National Institutes of Health, USA; Fremont Area Alzheimer's Fund, USA

**Research in context**

**Evidence before this study**

Extant literature from brain structure imaging and post-mortem neuropathology suggests that somatosensory neural systems (i.e., the brain systems supporting the sense of touch) are relatively unaffected by Alzheimer's disease (AD), along with other primary sensory systems. However, recent research has established a dynamic interplay between somatosensory neural systems and the brain systems supporting attention and executive functions. Patients with AD often have impaired attention and executive functions, but these impairments are highly variable across individuals, and so it is possible that controlling for variability in these cognitive abilities would reveal differences in somatosensory processing in these patients.

**Added value of this study**

This study investigates the usefulness of controlling for cognitive variability while testing for differences in somatosensory neural functions in patients with AD. The primary finding is that variability in attention (i.e., the ability to selectively focus cognitive resources) and processing speed (i.e., the time it takes to understand and react to incoming sensory information) abilities masks the detection of changes in somatosensory neural functions in patients with AD.

**Implications of all the available evidence**

These findings suggest that variability in cognitive declines, and in particular attention and processing speed, should be taken into account when testing for primary sensory impairments in patients with AD. Additionally, they suggest that when cognitive variability is properly controlled for, patients with AD exhibit robust changes in the neural processing of incoming somatosensory information.

Alt-text: Unlabelled box

## 1 Introduction

The pathological trajectory of Alzheimer's disease (AD) is now thought to begin as early as 20 years prior to the manifestation of hallmark declines in memory and other cognitive functions, beginning with the accumulation of amyloid-β “plaques” and fibrillary “tangles” rich in hyperphosphorylated tau [1,2]. Accompanying these early proteinopathies are measurable changes in the patterns of functional neuronal activity and structural neuronal morphology and integrity [1], [2], [3]. One of the most widely replicated findings in the AD neuroimaging literature is the sparing of primary sensory cortices, and particularly the primary sensorimotor cortices, until relatively late in the disease course [4], [5], [6], [7]. In addition, although the functional gating of redundant auditory information has been shown to be aberrant in patients with AD [8], [9], [10], [11], no such differences have been reported in the somatosensory domain. In combination with a sparsity of studies reporting neural somatosensory deficits in patients with AD [12], as well as others reporting null effects [7], this has led to a general consensus that somato-motor function is spared by the disease.

However, the functional neuroimaging literature has not considered a major confounding variable that is now known to robustly and systematically influence the functional processing of somatosensory stimuli: higher-order cognitive function. Patients with AD are known to exhibit variable declines in attention and processing speed [13,14], indicating that differing components of cognitive control are deficient at an early disease stage. These same cognitive functions are now known to interact with the neural processing of somatosensory stimuli [15], [16], [17], [18], [19], [20], [21], [22], as well as the functional gating of redundant somatosensory [23] and auditory [24], [25], [26], [27] information. Thus, it seems possible that unaccounted-for variability in attention and processing speed function might be masking functional aberrations in somatosensory processing in patients with AD. Supporting this possibility, previous findings of aberrant auditory processing in AD have been found to relate systematically to similar cognitive dysfunctions [10]. Further, a number of studies using animal models of AD, which do not exhibit the same degree of specialization and variability in executive functions as humans, have reported profound somatosensory aberrations [28,29].

In this study, we combine source imaged magnetoencephalography (MEG) during a paired-pulse somatosensory gating paradigm with an extensive series of neuropsychological assessments to determine whether variability in cognitive function masks somatosensory deficits in patients on the AD spectrum. MEG data were examined in the time and time-frequency domains at the sensor-level, and significant neural responses were source imaged. The relative response amplitude to somatosensory stimulation, as well as the gating of this sensory information between stimulation pairs was examined.

We hypothesized that attention and processing speed abilities, in particular, would play key roles in modelling differences in functional somatosensory activity between patients on the AD spectrum and a matched group of cognitively-normal older adults, while other cognitive faculties including memory, learning, and verbal function, would not.

## 2 Methods

### 2.1 Participants and ethics

The Institutional Review Board at the University of Nebraska Medical Center reviewed and approved this investigation (protocol #302-18-FB), and all research protocols complied with the Declaration of Helsinki. Written informed consent was obtained from each participant and, for participants in the AD spectrum group, from their spouse/child informant following a detailed description of the study. For individuals with diminished capacity to make an informed decision regarding research participation, educated assent was acquired from the participant, in addition to informed consent of their legally authorized representative. All participants completed the same experimental protocol. Exclusionary criteria for both groups included any medical illness affecting central nervous system function, any neurological disorder (other than Alzheimer's disease), history of head trauma, moderate or severe depression (Geriatric Depression Scale ≥ 10; [30]), and current substance abuse.

### 2.2 Alzheimer's disease spectrum group

Forty-four participants between 50 and 80 years of age, and without a history of psychiatric or other neurological disease (i.e., aside from amnestic mild cognitive impairment [MCI] or AD), were screened for recruitment into the AD spectrum group. These participants were initially referred to the study from the Memory Disorders Clinic at the University of Nebraska Medical Center (Omaha, NE, USA) and/or the Geriatric Medicine Clinic at Nebraska Medicine (Omaha, NE, USA) where they were being treated for amnestic memory complaints. Prior to being screened for this study all such participants were determined as having either aMCI or mild probable AD by a fellowship-trained neurologist using standard clinical criteria [31]. In addition to one of these diagnoses, a positive biomarker (using whole-brain quantitative amyloid-beta [Aβ] positron emission tomography [PET]) was also required for inclusion into the final AD spectrum participant sample. One participant was excluded from this group due to a major incidental finding that was likely to impact cognitive function, and another disenrolled due to COVID-19 related health concerns. Four additional participants were excluded after they were indicated as being amyloid-beta (Aβ) negative by means of whole-brain Aβ PET scanning with florbetapir ^18^F (see *Florbetapir ^18^F PET* below). After exclusions, 38 Aβ-positive participants remained for inclusion into the AD spectrum group.

### 2.3 Healthy aging comparison group

For comparison of the AD spectrum patients to a group of cognitively normal older adults, 20 additional participants who reported no subjective cognitive concerns were screened for inclusion into the study. Nineteen of these participants had received a biomarker test for Aβ-positivity within the past five years, and were confirmed biomarker-negative, while one participant received no such test, but performed exceedingly well on all neuropsychological examinations. Of note, the 19 amyloid-negative participants were recruited based on their previous enrollment in an unrelated clinical trial of an anti-amyloid drug in cognitively healthy older adults, where they were discovered to be amyloid-negative during the screening process and excluded from participation. These participants did not report cognitive disturbances, which was confirmed by our own detailed neuropsychological assessments (see *Methods: Neuropsychological Testing* and Table 1), and were not being seen at either of the clinics referenced above. Cognitively-normal participants were screened for inclusion in our study alongside the enrollment of the AD spectrum participants, and those who most closely matched the demographic makeup of the patient group were contacted. One participant was excluded from the comparison group for not completing the somatosensory experiment in its entirety, and one other for excessively noisy data (i.e., large movement/magnetic artifacts). After exclusions, 18 participants remained for inclusion into the cognitively-normal group.

**Table 1:** Demographics and neuropsychological profiles.

	Age (years)	Sex (% female)	Handedness (% left)		Education (years)				
**CN**	72.17 (4.66)	55.56	5.56		16.33 (2.89)				
**ADS**	69.21 (6.91)	47.37	7.89		15.50 (2.72)				
***p***	.106	.454	.751		.345				
	MoCA*	**MMSE**	**Learning**	**Memory**		**Attention**	**Verbal Fluency**		**Processing Speed**
**CN**	27.23 (1.92)	29.17 (1.10)	0.54 (0.75)	0.28 (0.56)		0.52 (0.61)	0.14 (0.78)		0.72 (0.83)
**ADS**	19.13 (4.76)	23.66 (4.15)	−2.04 (0.88)	−2.28 (0.70)		−0.77 (1.06)	−1.04 (1.01)		−0.90 (1.42)
***p***	<.001	<.001	<.001	<.001		<.001	<.001		<.001

Values reported are mean (SD), with p-values indicating significance of an unpaired t-test (continuous variables) or chi-square test (categorical variables). CN: cognitively normal; ADS: Alzheimer's disease spectrum; MoCA: Montreal Cognitive Assessment; MMSE: Mini-Mental State Exam.

⁎ *n* = 51 (MoCA scores were missing in five CN participants due to a data collection error).

Demographics for each group, as well as comparisons between groups, can be found in Table 1. Essential demographic factors were matched across the groups.

### 2.4 Florbetapir ^18^F PET acquisition and analysis

Combined PET/computed tomography (CT) data using ^18^F-florbetapir (Amyvid™, Eli Lilly) were collected following procedures described by the Society of Nuclear Medicine and Molecular Imaging (3D acquisition; single intravenous slow-bolus < 10 mL; dose = 370 MBq; waiting period = 30–50 min; acquisition = 10 min; [32]). A GE Discovery MI digital PET/CT scanner (Waukesha, WI) was used to acquire whole-brain quantitative images of amyloid-beta uptake. Images were attenuation-corrected using the CT data, and reconstructed in MIMNeuro (slice thickness = 2 mm; 33), converted to voxel-wise standardized uptake values (SUV) based on body weight, and then normalized into Montreal Neurological Institute (MNI) space. At this stage, each scan was read by a fellowship-trained neuroradiologist, who was blinded to their group assignment, and assessed as being “amyloid-positive” or “amyloid-negative” using established clinical criteria [33]. Patients who were amyloid-negative were excluded from the AD spectrum group. Images were then normalized to the crus of the cerebellum using the spatially unbiased infra-tentorial (SUIT) template [34] to generate voxel-wise maps of SUV ratios (SUVR; 2). Voxel-wise amyloid SUVRs were then extracted using peak somatosensory response coordinates from the grand-averaged gamma-frequency MEG responses (i.e., across both stimulations and all participants) and converted into MNI space using a non-linear transform [35]. These SUVR data were used to test hypothesized relationships with cognition and somatosensory function.

### 2.5 Neuropsychological testing

After screening and informed consent, participants completed a battery of neuropsychological assessments, with raw scores for each participant being converted to demographically-adjusted z-scores (e.g., based on age, education, etc.) using published normative data [36], [37], [38], [39]. This battery was developed in collaboration with a clinical neuropsychologist specializing in memory disorders, and focused on five cognitive domains generally impacted in patients with AD: *verbal memory* (Wechsler Memory Scale [WMS-IV] Logical Memory II Delayed Recall and Recognition [40]; Hopkins Verbal Learning Test-Revised [HVLT-R] Delayed Recall and Recognition Discriminability Index [39]), *learning* (WMS-IV Logical Memory I Recall [40]; HVLT-R Learning Trials 1-3 [39]), *attention and executive function* (Wechsler Adult Intelligence Scale [WAIS-IV] Digit Span Forward, Backward, and Sequencing [38]; Trail Making Test Part B [37]), *language* (Boston Naming Test [37]; Controlled Oral Word Association Test/Phonemic Verbal Fluency [37]; Animals/Semantic Verbal Fluency [37]), and *processing speed* (WAIS-IV Coding [38]; Trail Making Test Part A [37]). Demographically corrected z-scores based on test-specific normative data were averaged to create composite cognitive domain z-scores by participant. These domain composite scores were corroborated within the cognitively-normal group by calculating a ratio of z-scores representing the average of all correlations amongst intra-domain tests, divided by the average of all correlations with inter-domain tests. All domains had a ratio of z_intra_/z_inter_ > 1.40, and on average z_intra_/z_inter_ = 2.53 (SD = 1.51), indicating that these domains were ∼150% more internally- than externally-related.

### 2.6 Paired-pulse somatosensory paradigm

During the experiment, participants were seated with their eyes closed in a custom-made nonmagnetic chair with their head positioned within the MEG helmet-shaped sensor array. Unilateral electrical stimulation was applied to the right median nerve using external cutaneous stimulators connected to a Digitimer DS7A constant-current stimulator system (HW Medical Products, Neuberg, Germany). For each participant, at least 80 paired-pulse trials were collected using an inter-stimulus interval of 500 ms and an inter-pair interval that randomly varied between 4.3 and 4.8 s. Each pulse was comprised of a 0.2 ms constant-current square wave that was set to 10% above the motor threshold required to elicit a subtle twitch of the thumb.

### 2.7 MEG data acquisition

Our MEG data acquisition, structural coregistration, preprocessing, and sensor-/source-level analyses closely followed the analysis pipeline of previous manuscripts [23,[41], [42], [43], [44], [45]]. All recordings were conducted in a one-layer magnetically-shielded room with active shielding engaged. Neuromagnetic responses were sampled continuously at 1 kHz with an acquisition bandwidth of 0.1–330 Hz using a 306-sensor Elekta/MEGIN MEG system (Helsinki, Finland) equipped with 204 planar gradiometers and 102 magnetometers. Participants were monitored during data acquisition via real-time audio-video feeds from inside the shielded room. Each MEG dataset was individually corrected for head motion and subjected to noise reduction using the signal space separation method with a temporal extension (correlation limit: .950; correlation window duration: 6 s; [46]). Only data from the gradiometers were used for further analysis.

### 2.8 Structural MRI processing and MEG coregistration

Each participant's head position was monitored continuously throughout the recording, and the locations of the head position indicator coils were digitized, together with the three fiducial points and scalp surface (Fastrak 3SF0002, Polhemus Navigator Sciences, Colchester, VT, USA). Using these digitized points, each participant's MEG data were co-registered with their own structural T1-weighted magnetic resonance imaging (MRI) data using BESA MRI (Version 2.0) prior to source-space analysis. Structural MRI data were aligned parallel to the anterior and posterior commissures and transformed into standardized space. Following source analysis (i.e., beamforming), each participant's 4.0 × 4.0 × 4.0 mm functional images were also transformed into standardized space using the transform that was previously applied to the structural MRI volume and spatially resampled.

### 2.9 MEG preprocessing, time-frequency transformation, and sensor-level statistics

Cardiac and blink artifacts were removed from the data using signal-space projection (SSP), which was subsequently accounted for during source reconstruction [47]. The continuous magnetic time series was then filtered between 0.5–200 Hz plus a 60 Hz notch filter, and divided into 3700 ms epochs, with the baseline extending from −700 to −300 ms prior to the onset of the first somatosensory stimulus. Of note, we shifted our baseline away from the period immediately preceding stimulus onset to avoid potential contamination by any anticipatory responses. Epochs containing artifacts were rejected using a fixed threshold method, supplemented with visual inspection. Across all participants, the average amplitude threshold was 1231.77 (SD = 283.87) fT/cm, the average gradient threshold was 222.41 (SD = 131.10) fT/(cm*ms), and an average of 72.04 (SD = 11.53) trials (out of the original 80) were used for further analysis. Importantly, none of our statistical comparisons were compromised by differences in trial number nor artifact thresholds, as none of these metrics significantly differed across groups (Mann-Whitney U test; trial number: *p* = .628; amplitude threshold: *p* = .972; gradient threshold: *p* = .108). Note that a Mann-Whitney test was used here to account for the non-normal distribution of the number of accepted trials in the cognitively-normal group (Shapiro-Wilk test; *p =* .032) and of the gradient thresholds in both groups (Shapiro-Wilk test; AD spectrum: *p =* .002; cognitively-normal: *p* < .001).

To examine the phase-locked stimulus-evoked responses to somatosensory stimulation, the epochs remaining after artifact-rejection were averaged across trials to generate a mean time series per sensor, and the specific time windows used for subsequent source analysis were determined by statistical analysis of the sensor-level time series across both groups and the entire array of gradiometers. The time windows used for the source analysis were determined through paired-sample cluster-based permutation tests against baseline, with an initial cluster threshold of *p* < .001 and 10,000 permutations. The temporal windows of time-domain data that were non-exchangeable with baseline according to these permutation analyses were used to compute source images using standardized low resolution brain electromagnetic tomography (sLORETA; Tikhonov regularization constant: .01% of the leadfield matrix trace; [48]). The resulting whole-brain maps were 4-dimensional estimates of current density per voxel, per time sample. These data were normalized to the sum of the noise covariance and theoretical signal covariance, and thus the units are arbitrary. Using the two temporal clusters identified in the sensor-level analysis (see below), these maps were averaged over time and across groups. The resulting maps were then averaged across the two windows to determine the peak voxel of the time-domain neural response to the stimuli across participants. From this peak, the sLORETA units were extracted to derive estimates of each time-domain response amplitude per participant.

In order to also examine the role of band-limited neural responses to somatosensory stimuli, we transformed the post-artifact-rejection sensor-level epochs into the time-frequency domain using complex demodulation [49], [50], [51]. The time-frequency analysis was performed with a frequency-step of 2 Hz and a time-step of 25 ms between 4 and 100 Hz, using a 4 Hz lowpass finite impulse response (FIR) filter with a full-width half maximum in the time domain of ∼115 ms. The resulting spectral power estimations per sensor were averaged over trials to generate time-frequency plots of mean spectral density, which were normalized by the baseline power of each respective bin ((active-baseline)/baseline), calculated as the mean power during the −700 to −300 ms time period. The time-frequency windows used for the source analysis were again determined by means of a paired-sample cluster-based permutation test against baseline across all participants and the entire frequency range (4–100 Hz), with an initial cluster threshold of *p* < .001 and 10,000 permutations.

Time-frequency resolved beamformer source images were computed with the dynamic imaging of coherent sources approach (DICS; truncated singular value decomposition [SVD] regularization: values <.0001% of maximum SVD set to zero; 49, [52]). Following convention, we computed noise-normalized, source power per voxel in each participant using active (i.e., task) and passive (i.e., baseline) periods of equal duration and bandwidth. This approach generated three-dimensional participant-level pseudo-t maps per each time-frequency cluster identified in the sensor-level analysis. These voxel-wise maps of oscillatory neuronal response amplitude were averaged within groups across the two time windows identified through the statistical analysis, corresponding to stimulations 1 and 2, for display purposes and then grand-averaged across both stimulations and all participants. The voxel of maximum amplitude was then identified from this grand-averaged map, and virtual sensor data were extracted from this grand-average peak voxel. This signal was decomposed into time-frequency space, baseline-corrected, and was then averaged across the time and/or frequency ranges identified through the sensor-level analyses for statistical testing and visualization of the neural amplitude envelope.

For each neural response, relative amplitude values for stimulations 1 and 2 were averaged within each participant to represent the average neural response to somatosensory stimulation, and somatosensory gating ratios were derived using the following formula: SG = (A_stim2_ + 2)/(A_stim1_ + 2), where A is the relative response amplitude and SG is the gating ratio. The addition of a constant to each stimulation amplitude nulled any spurious effects of near-zero or negative values on the estimation of the gating ratio (no constant was added to the sLORETA values, which are always >0). Participants exhibiting outlier average neural response amplitudes or somatosensory gating ratios, as determined by a fixed threshold of ±2 standard deviations from the mean, were excluded listwise per each neural response from all statistical models involving that response. Differences between the groups in major demographic factors remained unchanged after these exclusions.

### 2.10 Statistical analysis and software

Since group differences in age approached significance, all statistical analyses were performed including age as a nuisance covariate. Per each neural response of interest, general linear models were initially computed to test for group differences in the average somatosensory neural responses and gating ratios. Next, the cognitive domain composite scores for attention and processing speed (see *Neuropsychological* Testing) were added individually into each of these two models (i.e., one for group differences in response amplitude and another for group differences in gating ratio) as a covariate of interest, to determine which cognitive domains significantly contributed to prediction of the dependent variable. Differences in model predictive value (ΔR^2^) between the models with and without the cognitive scores included were also computed. Cognitive domain composite scores that fulfilled the basic requirements of mediation/suppression [53,54] were tested for indirect effects on the groupwise differences in somatosensory function using a nonparametric bootstrapping approach with 10,000 simulations [55]. Relationships between cognition and amyloid-β uptake were probed using general linear models that regressed neuropsychological domain scores on SUVRs from the MEG somatosensory peak voxel, above and beyond the effects of age. All MEG data preprocessing, coregistration, and sensor- and source-level analyses were performed in the Brain Electrical Source Analysis software suite (BESA Research v7.0 and BESA MRI v2.0). Cluster-based permutation testing on MEG sensor-array data was performed in BESA Statistics (v2.0). General linear models were computed using the *stats* package in *R* [56], and indirect effects were evaluated using the *mediation* package [55]. Plotting of model residuals used *ggplot2* [57].

### 2.11 Role of funding source

The funders had no role in the study design, data collection, data analyses, interpretation, or writing of this report.

## 3 Results

**Fig. 1:**
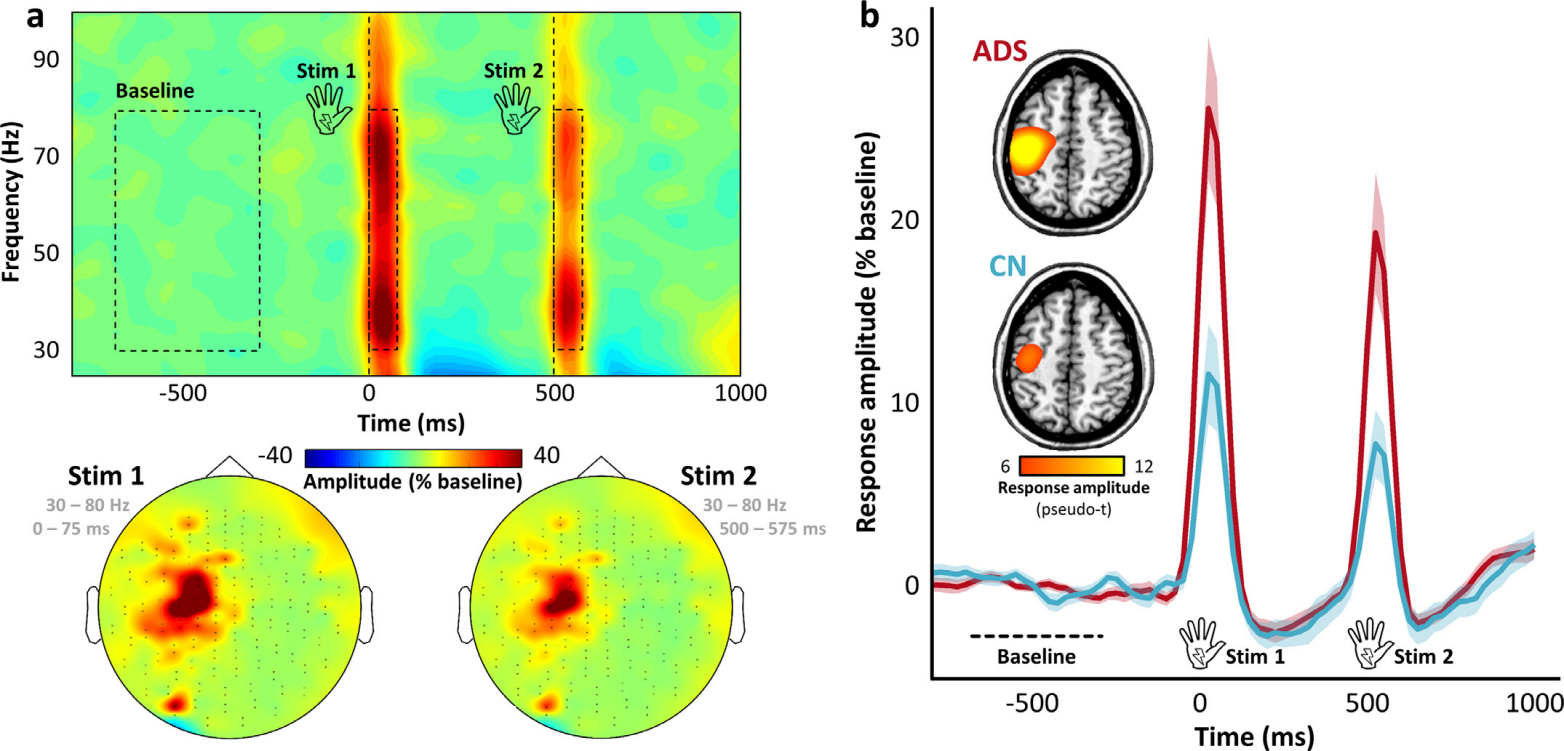
Oscillatory neural responses to paired-pulse somatosensory stimulation. (**a**) The spectrogram (top) displays time-frequency data from a representative gradiometer (MEG0233), with time represented (in milliseconds) on the x-axis and frequency represented (in Hz) on the y-axis. The two vertical dotted lines represent the onset of the paired-pulse somatosensory stimulations (at 0 and 500 ms), and the time-frequency windows used as the pre-stimulus baseline and those identified as the neural responses to somatosensory stimulation in the sensor-level statistical analysis, are outlined by black dotted rectangles. The topographic maps (bottom left) indicate the spatial distribution of the gamma-frequency (30–80 Hz) responses to first (left; 0–75 ms) and second (right; 500–575 ms) somatosensory stimulations. The color bar in the middle of the figure displays the amplitude thresholds (in percent change from baseline) used for display of both the spectrogram and the topographic maps. (**b**) Inlaid brain images indicate the source-imaged data, averaged over both somatosensory responses and within each group (ADS: Alzheimer's disease spectrum, red; CN: cognitively-normal, blue), with the amplitude thresholds (in pseudo-t values) used for display shown on the color bar below. The time series represent peak-voxel amplitude envelopes for these gamma responses, per each group, with time (in milliseconds) on the x-axis and response amplitude (in percent change from baseline) on the y-axis. Shaded areas indicate ± 1 standard error of the mean. The baseline interval, as well as the onset of each somatosensory stimulation, are indicated just above the x-axis.

Robust neural responses to each somatosensory stimulation were identified in the time-frequency domain in the theta (4–8 Hz; 25–75 ms post-stimulus), alpha (8–12 Hz; 225–425 ms post-stimulus), beta (16–24 Hz; 100–350 ms post-stimulus), and gamma (30–80 Hz; 0–75 ms post-stimulus) bands (Figs. 1a and S1; cluster-based permutation test; 10,000 permutations; *p* < .001). Additionally, a significant somatosensory evoked response extended from 25 to 170 ms post-stimulus in the time domain (cluster-based permutation test; 10,000 permutations; *p* < .001). Source imaging of these responses indicated that they all originated from the hand-knob region of the postcentral gyrus (Figs. 1 and S1–S2), suggesting somato-motor origin.

### 3.1 Variability in processing speed abilities masks increased gamma somatosensory responses in patients on the AD spectrum

**Fig. 2:**
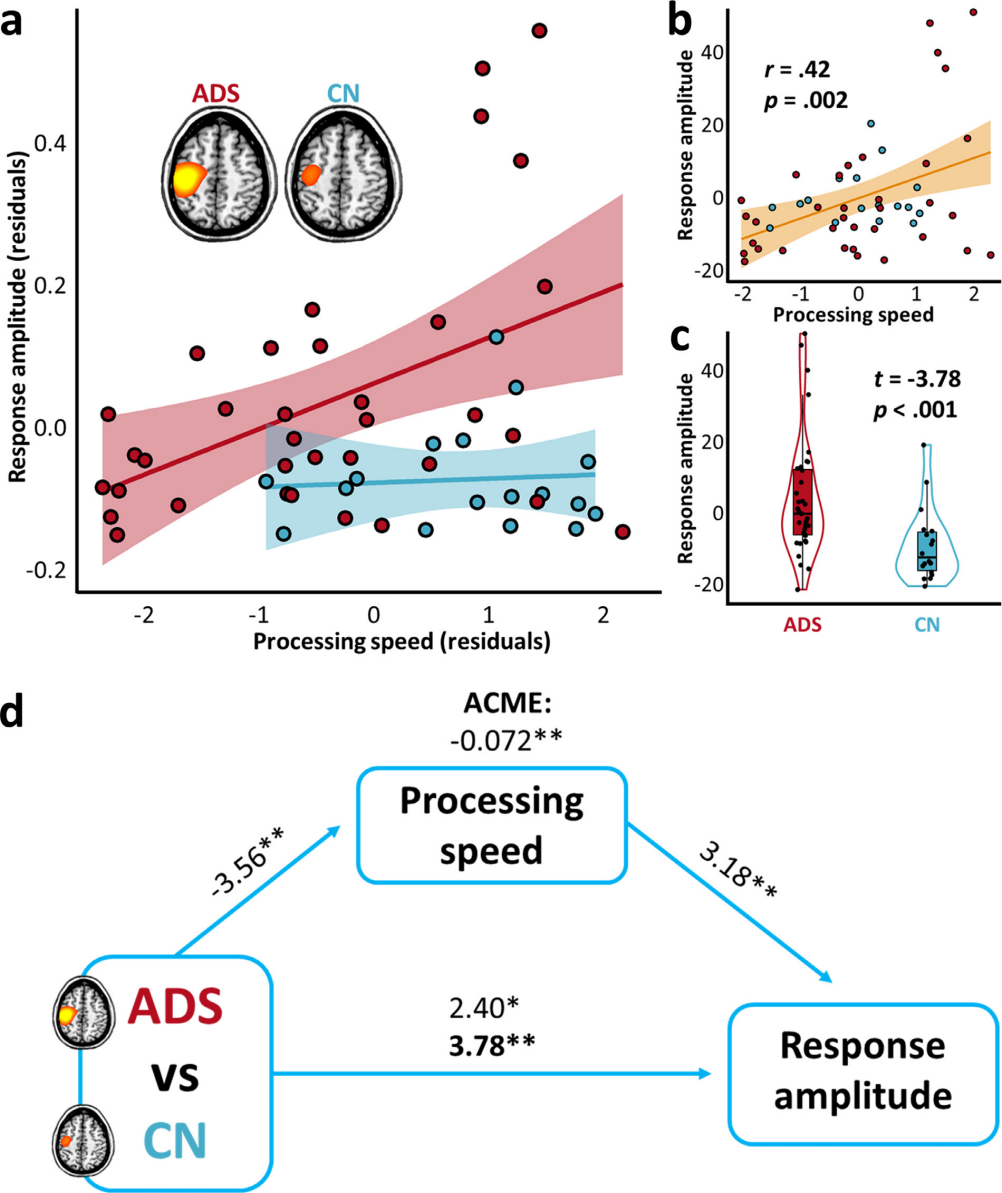
Processing speed abilities suppress group differences in somatosensory response amplitude. The scatterplot in (**a**) indicates the relationship between processing speed abilities (x-axis) and response amplitude (y-axis), per each group (ADS: Alzheimer's disease spectrum, red; CN: cognitively-normal, blue), above and beyond the effects of age. The lines of best fit, per each group, are overlaid with 95% confidence intervals indicated in the shaded area. The scatterplot in (**b**) indicates the same relationship, above and beyond the effects of age and group, with the partial correlation coefficient **(r)** and corresponding p-value overlaid, along with the line of best-fit and 95% confidence intervals. The plot in (**c**) represents the difference in response amplitude as a function of group, above and beyond the effects of processing speed and age, with the t-value and corresponding p-value overlaid. Box plots represent conditional means, first and third quartiles, and minima and maxima, and violin plots show the probability density. Paths in (**d**) between the three variables of interest are represented by blue arrows, with t-values above each indicating the relationship strength, above and beyond the effects of age. The bold t-value at the bottom represents the relationship between group and response amplitude, after accounting for the effect of processing speed scores, and the average causal mediation effect (ACME) at top represents the indirect impact of processing speed on this relationship (10,000 bootstrapping simulations). **p < .005. *p < .05.

A general linear model of group differences in gamma-frequency somatosensory response amplitude (i.e., averaged over the two stimulations) initially indicated a significant, but weak, increase in amplitude in patients on the AD spectrum (*F*(1,49) = 5.78, *p =* .020). Accounting for variability across participants in processing speed scores significantly enhanced the overall model (Fig. 2a,c; Δ*R^2^ =* .16, *p =* .003), and significantly increased the model sensitivity to group differences in gamma response amplitude via an indirect effect (Fig. 2d; causal mediation analysis; average causal mediation effect [ACME] = −.07, *p =* .004; average direct effect [ADE] = .19, *p* < .001; proportion mediated = −.62, *p =* .005). The addition of no other cognitive domain scores, including attention, learning, memory, and verbal function, significantly contributed to model accuracy (general linear model; all *p*’s > .05).

### 3.2 Variability in attention and processing speed masks increased gamma-frequency somatosensory gating in patients on the AD spectrum

**Fig. 3:**
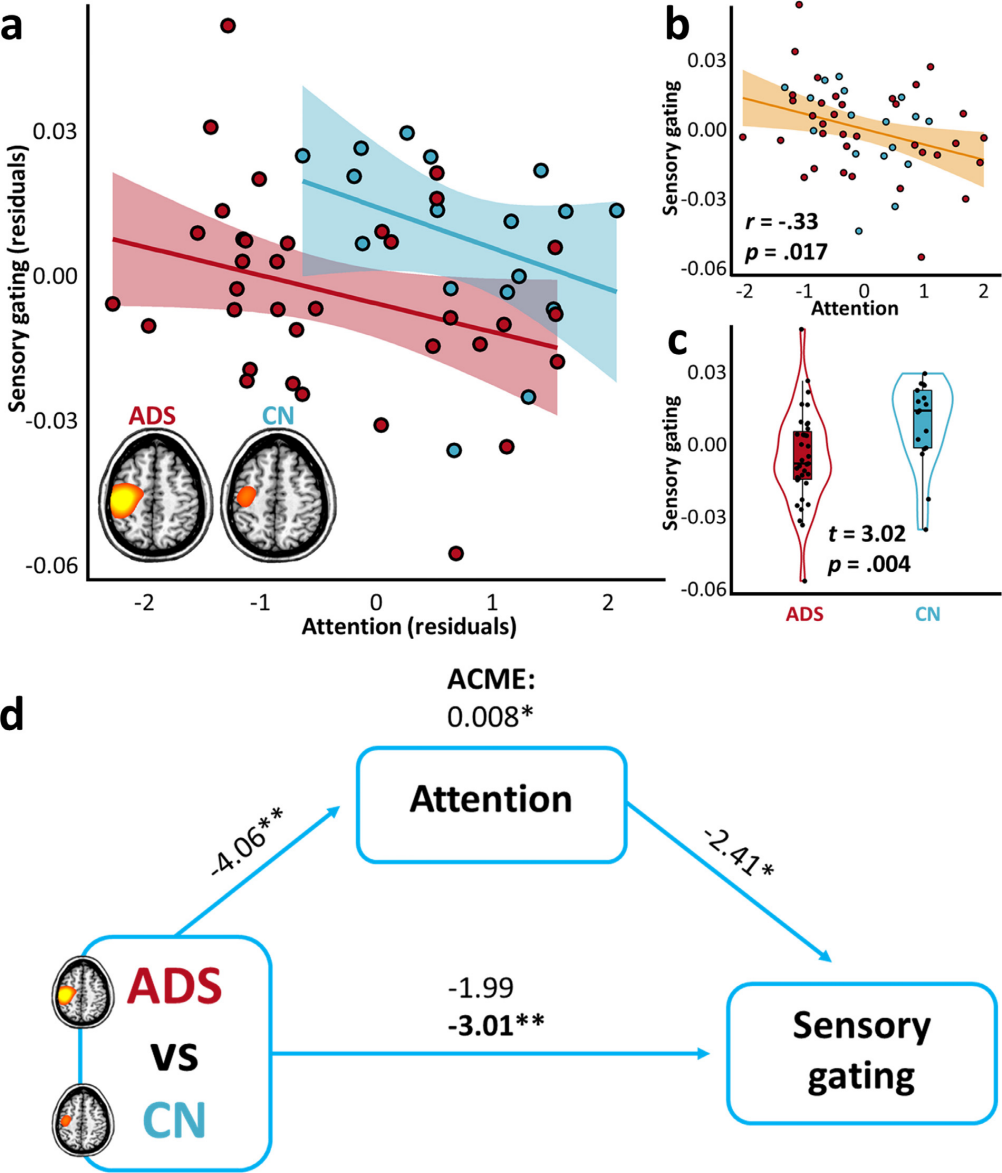
Attention abilities suppress group differences in somatosensory gating. The scatterplot in (**a**) indicates the relationship between attention abilities (x-axis) and the somatosensory gating ratio (y-axis), per each group (ADS: Alzheimer's disease spectrum, red; CN: cognitively-normal, blue), above and beyond the effects of age. The lines of best fit, per each group, are overlaid with 95% confidence intervals indicated in the shaded area. The scatterplot in (**b**) indicates the same relationship, above and beyond the effects of age and group, with the partial correlation coefficient **(r)** and corresponding p-value overlaid, along with the line of best-fit and 95% confidence intervals. The plot in (**c**) represents the difference in somatosensory gating as a function of group, above and beyond the effects of attention and age, with the t-value and corresponding p-value overlaid. Box plots represent conditional means, first and third quartiles, and minima and maxima, and violin plots show the probability density. Paths in (**d**) between the three variables of interest are represented by blue arrows, with t-values above each indicating the relationship strength, above and beyond the effects of age. The bold t-value at the bottom represents the relationship between group and somatosensory gating, after accounting for the effect of attention scores, and the average causal mediation effect (ACME) at the top represents the indirect impact of attention on this relationship (10,000 bootstrapping simulations). **p < .005. *p < .05.

**Fig. 4:**
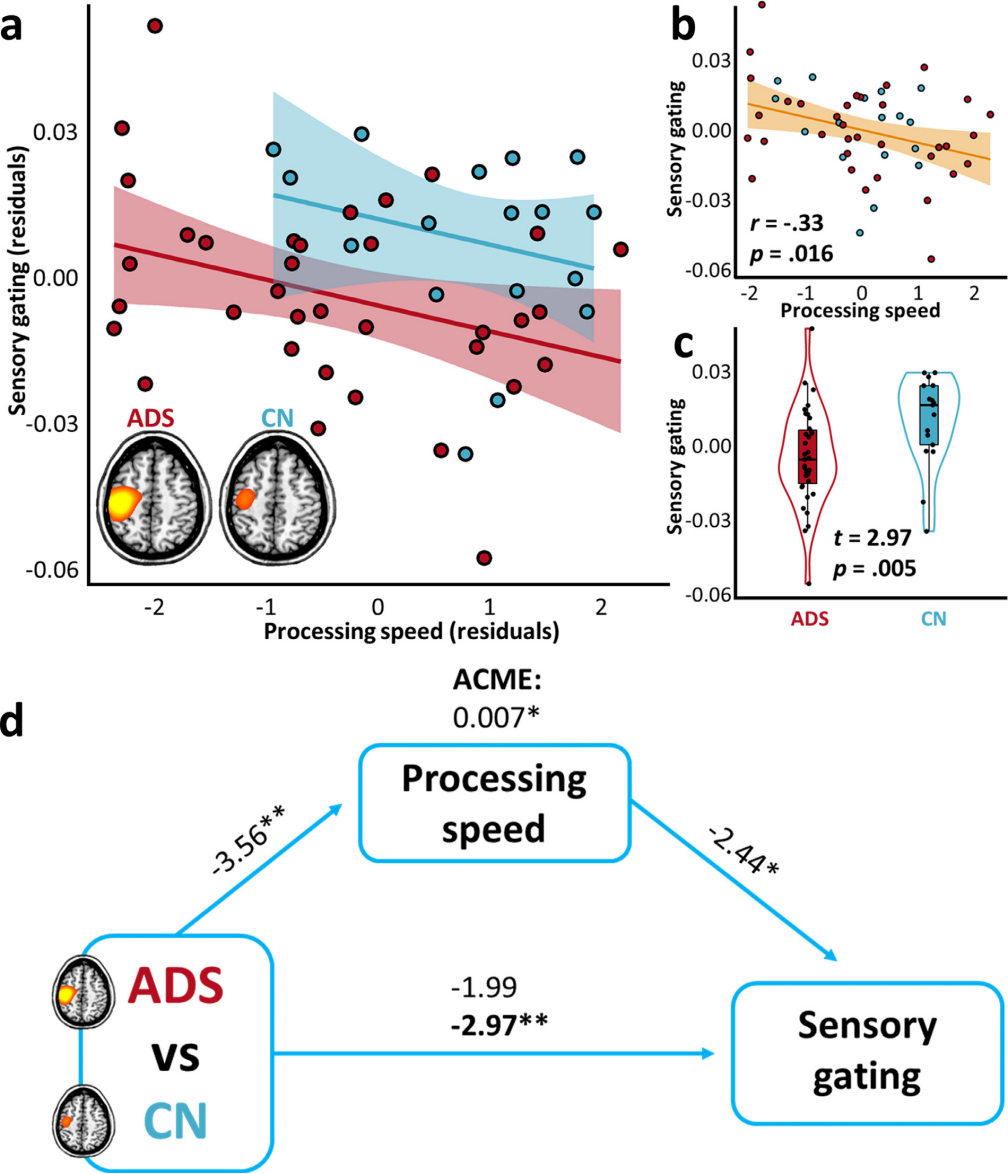
Processing speed abilities suppress group differences in somatosensory gating. The scatterplot in (a) indicates the relationship between processing speed abilities (x-axis) and the somatosensory gating ratio (y-axis), per each group (ADS: Alzheimer's disease spectrum, red; CN: cognitively-normal, blue), above and beyond the effects of age. The lines of best fit, per each group, are overlaid with 95% confidence intervals indicated in the shaded area. The scatterplot in (b) indicates the same relationship, above and beyond the effects of age and group, with the partial correlation coefficient (r) and corresponding p-value overlaid, along with the line of best-fit and 95% confidence intervals. The plot in (c) represents the difference in somatosensory gating as a function of group, above and beyond the effects of processing speed and age, with the t-value and corresponding p-value overlaid. Box plots represent conditional means, first and third quartiles, and minima and maxima, and violin plots show the probability density. Paths in (d) between the three variables of interest are represented by blue arrows, with t-values above each indicating the relationship strength, above and beyond the effects of age. The bold t-value at the bottom represents the relationship between group and somatosensory gating, after accounting for the effect of processing speed scores, and the average causal mediation effect (ACME) at the top represents the indirect impact of processing speed on this relationship (10,000 bootstrapping simulations). **p < .005. *p < .05.

Next, a linear model was computed to examine group differences in the functional gating of gamma-frequency somatosensory responses. Again, the initial model (i.e., with no consideration for cognitive domain scores) indicated a weak, and this time non-significant, decrease in the somatosensory gating ratio in patients on the AD spectrum (*F*(1,49) = 3.96, *p =* .052). The addition of attention domain scores to this model significantly enhanced both the overall model (Fig. 3a–c; Δ*R^2^ =* .10, *p =* .020), as well as the sensitivity of the model to group differences in somatosensory gating (Fig. 3d; causal mediation analysis; ACME = .01, *p =* .009; ADE = −.02, *p =* .003; proportion mediated = −.67, *p =* .047). Considering between-participant variability in processing speed scores also provided enhanced model accuracy (Fig. 4a–c; Δ*R^2^ =* .10, *p =* .019), and improved prediction of group differences in somatosensory gating ratios (Fig. 4d, bottom; causal mediation analysis; ACME = .01, *p =* .016; ADE = −.02, *p =* .003; proportion mediated = −.59, *p =* .048). The addition of no other cognitive domain scores, including learning, memory, or verbal function, significantly contributed to model accuracy (all *p*’s > .05).

No models of the theta, alpha, beta, or evoked responses indicated a potential suppression of group differences in somatosensory metrics by cognitive scores (Table S1). However, there was a significant difference in the gating of the alpha somatosensory response (*F*(1,48) = 4.75, *p =* .034), such that the gating ratio was reduced in patients on the AD spectrum. Importantly, since the alpha response was a *decrease* from resting levels of synchrony, this indicates that the gating of this somatosensory response was reduced in patients compared to their demographically-matched counterparts.

### 3.3 Local amyloid-β uptake in primary somatosensory cortex predicts learning abilities in patients on the AD spectrum

**Fig. 5:**
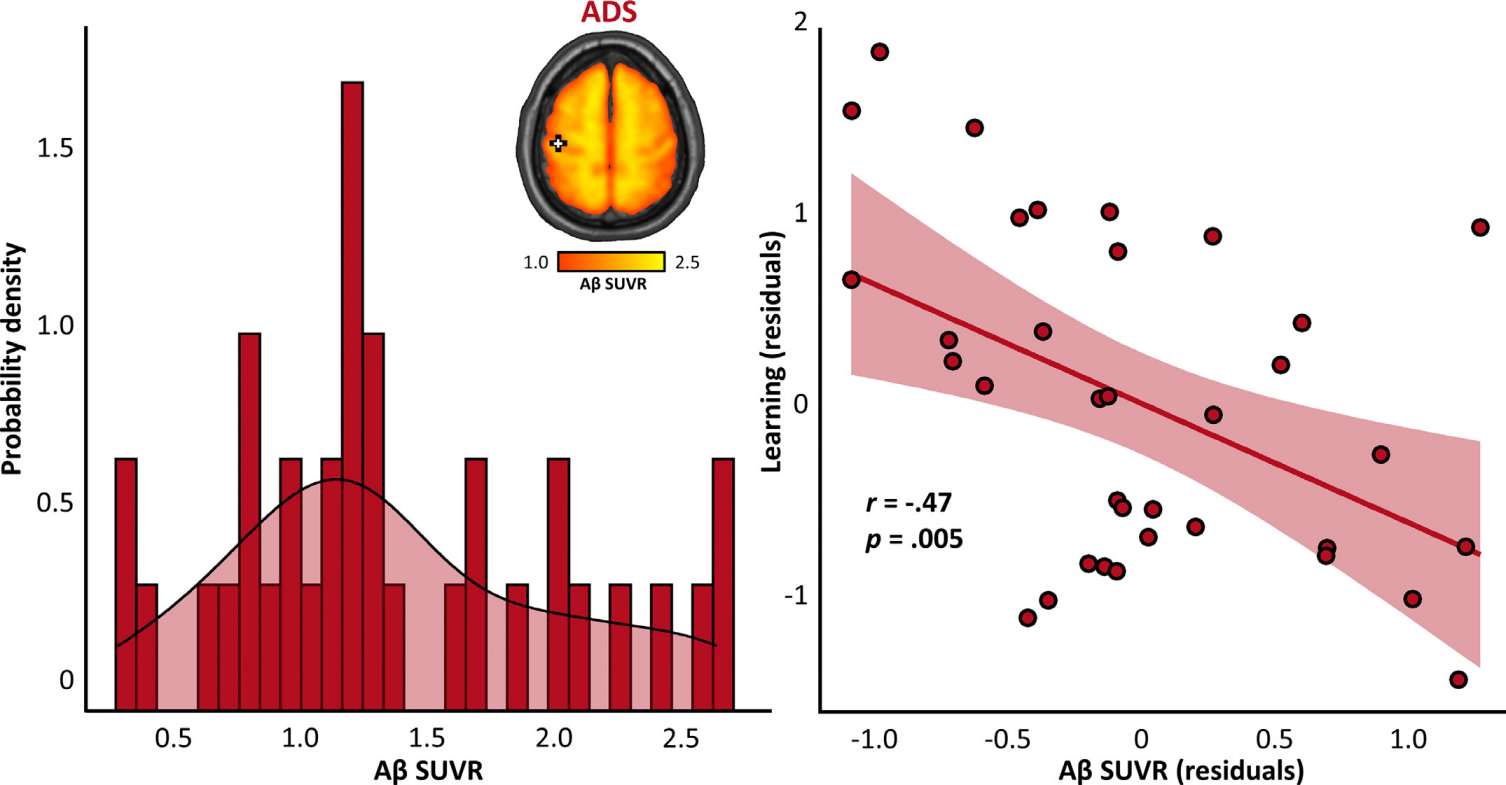
Amyloid-β uptake in primary somatosensory cortex predicts learning in patients on the AD spectrum. The histogram and density plot on the left indicates the distribution of amyloid-β SUVRs (x-axis) at the peak voxel of the MEG somatosensory response in patients on the AD spectrum (shown on the inlaid PET image). The scatterplot on the right indicates the relationship between this somatosensory amyloid-β uptake (in SUVRs; x-axis) and learning function (y-axis) in patients on the AD spectrum, above and beyond the effects of age. The line of best fit is overlaid with 95% confidence intervals indicated in the shaded area, as are the partial correlation coefficient and corresponding p-value.

Finally, to determine whether proteinopathy in primary somatosensory cortex was related to cognitive function in patients on the AD spectrum, we extracted SUVRs from the peak voxel identified in the MEG gamma-frequency analysis and regressed these data on each of the five cognitive domain scores. We found a significant relationship between learning domain scores and amyloid-β SUVRs, such that increased amyloid-β pathology in primary somatosensory cortex predicted worse learning performance (partial correlation; *r*(33) = -.47, *p =* .005; Fig. 5). No such effect was observed for memory (*r*(33) = −.31, *p =* .075), verbal function (*r*(33) = −.22, *p =* .222), processing speed (*r*(33) = −.18, *p =* .315), or attention (*r*(33) = −.30, *p =* .086). Of note, this local proteinopathy did not significantly predict the relative amplitude of somatosensory responses (*r*(33) = .17, *p =* .339), nor the functional gating of redundant somatosensory information (*r*(33) = −.12, *p =* .494).

## 4 Discussion

Despite an extensive literature reporting somatosensory dysfunction in animal models of AD [28,29], very little evidence exists supporting such pathology in human patients. While this discrepancy might be partially attributable to inherent differences in the AD neuropathology of humans and that observed in animal models, we find evidence that it might instead stem from a robust suppression effect attributable to between-participant variability in cognitive function in AD, explicitly in two key domains: attention and processing speed. Initial models of functional differences in gamma-frequency somatosensory processing between patients on the AD spectrum and cognitively-normal older adults showed only weak and marginally-significant effects. However, the addition of attention and processing speed cognitive scores to these models revealed pronounced somatosensory dysfunction in these patients, indicating a suppression effect that was substantiated using a bootstrapping approach for significant indirect effects. Specifically, between-participant variability in processing speed abilities masked a substantial increase in the amplitude of the neural gamma band response to somatosensory stimulation, while similar variability in both attention and processing speed masked a considerable increase in the functional gating of redundant somatosensory information (i.e., hyper-gating) of the same neural response. These findings mark one of the first studies to report key differences in somatosensory processing in patients on the AD spectrum, while also providing a theoretical mechanism for the notable lack of such findings in extant literature.

The practical implications of these findings are twofold. First, and perhaps most importantly, the robust suppression effects that we report here indicate that the fidelity of neural processing of primary somatosensory stimuli in patients with AD is dependent on inter-individual variabilities in attentional and processing speed. This dependency should be taken into account in future studies of primary sensory function more broadly to better understand the nuanced effects of AD on, for example, visual and auditory processing. This appears to be particularly important for the study of high-frequency oscillations, as we found no evidence for suppression of the evoked, theta, alpha, or beta responses by cognitive variability. Second, this is one of the first reports of pronounced differences in cortical somatosensory processing in people with AD, and to our knowledge, the first report of somatosensory gating deficits in this patient group. These findings should guide future studies on the mechanistic bases of this effect. For example, using a previously reported approach [23] to systematically interrogate the influence of directed attention on somatosensory gating in these patients would be useful to determine the inter-regional functional connections that potentially mediate AD-related changes in somatosensory gating.

Deficits in attention and processing speed are both well documented in patients with AD [13,14], however the degree of such impairments often vary considerably across individuals, as was the case in our sample. In contrast, reports of somatosensory dysfunction in this patient group are exceedingly rare, with the notable exception of a study that used MEG to examine somatosensory event-related potentials in patients with mild cognitive impairment and AD [12]. In this study, Stephen et al. found a modest but significant increase in somatosensory response amplitude in patients with mild cognitive impairment, but no somatosensory gating effects were explored. Interestingly, post-hoc exploration of their data revealed that performance on neuropsychological tests may have had a qualitative suppression effect on this difference, however this possibility was not tested empirically. Although highly informative, this study did not restrict the participant sample to biomarker-confirmed patients, and did not comprehensively evaluate the role of distinct neuropsychological domains to such suppression effects, limiting possible interpretations. Our findings extend this literature substantially, by showing that both the relative response amplitude to stimulation and the functional gating of redundant somatosensory information are increased in patients on the AD spectrum when cognitive function is considered in parallel. In other words, when cognitive function is taken into account, patients on the AD spectrum exhibit abnormally stronger somatosensory responses and more robust gating (i.e., hyper-gating).

Somatosensory function has been found to be impaired in a number of other neurological, developmental, and psychiatric disorders, including Parkinson's disease [58], HIV-associated neurocognitive disorders [42,[59], [60], [61]], autism [62], cerebral palsy [63, 64], schizophrenia [65], and others. Typically, such deficits are indicated by a reduced neural response to somatosensory stimuli, however, our findings indicate the inverse in AD: a hyper-sensitivity to stimulation. This aligns with previous studies that used transcranial magnetic stimulation (TMS) to examine motor cortex excitability in patients with AD, and found that weaker stimulation was required to elicit a similar motor-evoked potential, indicating hyper-excitability of the motor cortex [66,67]. Such motor hyper-excitability has been linked to impaired high-frequency glutamatergic neurotransmission in local excitatory intracortical circuits [66,68], and thus it seems likely that similar mechanisms underlie the high-frequency somatosensory excitability that we find in the current study. Our novel finding of a robust pattern of stronger somatosensory gating in patients on the AD spectrum is in stark contrast to previous reports of *reduced* auditory gating in AD [8], [9], [10], [11]. Interestingly, this decreased gating of redundant auditory information in AD has been shown to also be mediated by attention function [10], indicating a bi-directional interplay between cognitive variability and sensory gating in the auditory and somatosensory domains in AD. Importantly, hyper-gating of redundant stimuli is not necessarily beneficial, as children with cerebral palsy exhibit a similar phenomenon in high-frequency somatosensory responses [63].

### 4.1 Caveats and limitations

Although our findings are of interest, the limitations of this study should also be acknowledged. We investigated the potential for an influence of local amyloid-β accumulation on these patterns of somatosensory neural dysfunction, and found no evidence for such an effect. However, we did find a significant relationship between somatosensory amyloid-β SUVRs and learning. This indicates that somatosensory amyloidopathy, which occurs relatively late in the course of AD, scales with cognitive declines in at least one domain. Although fascinating, a more comprehensive approach that leverages both tau and amyloid-β imaging, preferably in preclinical stages of the disease, is necessary to validate and extend this finding. Further, studies that leverage the higher inter-regional variability in amyloid-β burden within patients (i.e., as opposed to inter-participant variability within a single cortical region) might be more sensitive to other relationships between amyloidopathy and somatosensory neural dynamics. Despite being at or above the levels of previous studies in this field [69,70], our participant sample sizes were also relatively modest. Although our inclusion/exclusion criteria of a positive amyloid biomarker and subjective amnestic complaint for the patient group, and the absence of both for the cognitively-normal controls, were essential in reducing variability within our participant groups, substantial variability in clinical and cognitive metrics within the groups was still quite apparent. Substantiating the effects reported here in larger participant groups is essential to enhance generalizability. This is particularly important given that AD is a highly heterogeneous disorder, and so examining these effects in a larger patient sample might also allow for generalization to sub-types of AD, which are recognized as meaningfully distinct [71], [72], [73].

## 5 Conclusion

In conclusion, the differences in the neural dynamics observed here indicate that, although masked by highly variable between-participant declines in cognitive function, neural processing in the primary somatosensory cortices is strongly impacted by AD. Further, the robust suppression effects on such differences by attention and processing speed abilities in these patients indicate that potential cognitive mediators should be better modeled and controlled in the AD neuroimaging literature.

## Contributors

Alex I Wiesman: Conceptualization, Methodology, Software, Formal Analysis, Investigation, Resources, Writing – Original Draft, Visualization, Supervision, Project Administration, Funding Acquisition. Victoria M Mundorf: Formal Analysis, Writing – Review & Editing. Chloe C Casagrande: Formal Analysis, Writing – Review & Editing. Sara L Wolfson: Resources, Writing – Review & Editing. Craig M Johnson: Methodology, Investigation, Resources, Writing – Review & Editing. Pamela E May: Conceptualization, Methodology, Resources, Writing – Review & Editing. Daniel L Murman: Conceptualization, Methodology, Resources, Writing – Review & Editing. Tony W Wilson: Conceptualization, Methodology, Resources, Writing – Review & Editing, Supervision, Funding Acquisition. All authors have read and approved the final version of this manuscript. Authors AIW, VMM, CCC, and TWW have verified the underlying data.

## Data sharing statement

The data that support the findings of this study are available from the corresponding author, Dr. Alex I. Wiesman, upon reasonable request.

## Declaration of Competing Interest

All authors declare no conflicts of interest. Dr. Murman reported receiving grants from Green Valley Pharmaceuticals, Functional Neuromodulation, Roche, and Eli Lilly and Co. and serving on an advisory board for Biogen. Dr. Wilson reported serving as a board member for the American Clinical Magnetoencephalography Society and the International Society for the Advancement of Clinical Magnetoencephalography.

## Appendix Supplementary materials

Image, application 1

Image, application 2
