# Supplementary material for: Somatosensory dysfunction is masked by variable cognitive deficits across patients on the Alzheimer's disease spectrum
Source: eBioMedicine. 2021 Oct 21;73:103638. doi: 10.1016/j.ebiom.2021.103638 (PMC8550984; doi:10.1016/j.ebiom.2021.103638)
Supplement: Supplementary file 1 [file mmc1.pdf]

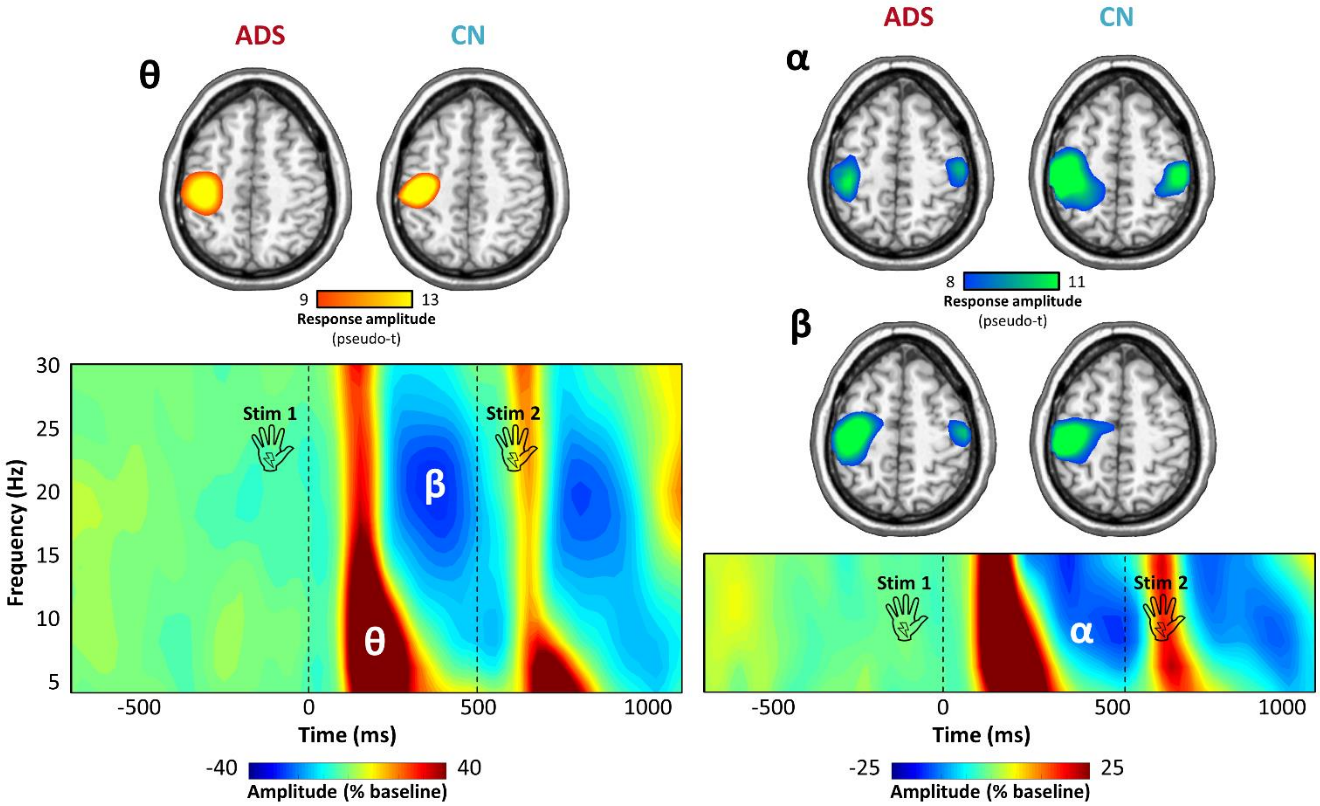

Figure S1. Somatosensory responses in the theta, alpha, and beta bands. The spectrograms (bottom) display time-frequency data from representative gradiometers (left: MEG0233; right: MEG1812), with time represented (in milliseconds) on the x-axes and frequency represented (in Hz) on the y-axes. The vertical dotted lines represent the onset of the two somatosensory stimulations (at 0 and 500 ms), and the time-frequency responses identified in the sensor-level analysis are indicated by greek letters. Brain images (top) indicate the source-imaged data, averaged over both somatosensory responses and within each group (ADS: Alzheimer's disease spectrum, red; CN: cognitively-normal, blue), with the amplitude thresholds (in pseudo-t values) used for display shown on the respective color bars.

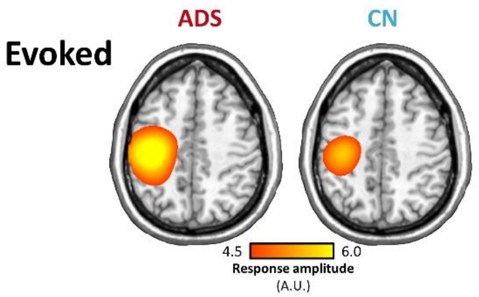

Figure S2. Somatosensory evoked responses in the time domain. Brain images indicate the source-imaged evoked somatosensory response, averaged over both stimulations within each group (ADS: Alzheimer’s disease spectrum [left]; CN: cognitively-normal [right]), with the amplitude thresholds (in arbitrary units, A.U.) used for display shown on the color bar below.

Table S1. Model summary statistics for each somatosensory response.

Response Amplitude	Peak Location (x, y, z)			ADS vs CN (t-value)	Attention Effect (ΔR^2)	Attention ACME	Processing Speed Effect (ΔR^2)	Processing Speed ACME
Theta (4 – 8 Hz)	-38	-27	49	-1.16	.016	-	.013	-
Alpha (8 – 12 Hz)	-43	-26	44	-0.20	.061#	-	.081*	-
Beta (16 – 24 Hz)	-38	-23	53	-0.44	.008	-	.029	-
Gamma (30 – 80 Hz)	-42	-23	53	2.40*	.027	-	.155**	-.072**
Evoked	-38	-23	48	1.83#	.004	-	.003	-
**Somatosensory Gating**	**Peak Location (x, y, z)**			**ADS vs CN (t-value)**	**Attention Effect (ΔR^2)**	**Attention ACME**	**Processing Speed Effect (ΔR^2)**	**Processing Speed ACME**
Theta (4 – 8 Hz)	-38	-27	49	-1.50	.007	-	.029	-
Alpha (8 – 12 Hz)	-43	-26	44	-2.18*	.052#	-	.032	-
Beta (16 – 24 Hz)	-38	-23	53	0.30	.024	-	.017	-
Gamma (30 – 80 Hz)	-42	-23	53	-1.99#	.099*	.008*	.101*	.007*
Evoked	-38	-23	48	-1.89#	.014	-	.002	-

#$p < .10$, *$p < .05$, **$p < .005$. ACME: average causal mediation effect. Peak voxel coordinates are defined in MNI space.
